# Daily rhythm in DNA methylation and the effect of total sleep deprivation

**DOI:** 10.1111/jsr.14438

**Published:** 2024-12-15

**Authors:** Antti‐Jussi Ämmälä, Thomas P. M. Hancox, Fan Qiuyu, Alexandra Lahtinen, Sonja Sulkava, Victoria L. Revell, Katrin Ackermann, Manfred Kayser, Debra J. Skene, Tiina Paunio

**Affiliations:** ^1^ Sleepwell Program and Department of Psychiatry University of Helsinki and Helsinki University Hospital Helsinki Finland; ^2^ Department of Public Health and Welfare, Population Health Unit National Institute of Health and Welfare Helsinki Finland; ^3^ Terveystalo Plc, Medical Lead Helsinki Finland; ^4^ School of Biosciences, University of Birmingham Birmingham UK; ^5^ Department of Clinical Genetics Helsinki University Hospital Helsinki Finland; ^6^ Research Program in Systems Oncology, Research Programs Unit, Faculty of Medicine, University of Helsinki Helsinki Finland; ^7^ Surrey Sleep Research Centre, Faculty of Health and Medical Sciences, University of Surrey Guildford UK; ^8^ EaStCHEM School of Chemistry, Biomedical Sciences Research Complex, and Centre of Magnetic Resonance, University of St Andrews St Andrews UK; ^9^ Department of Genetic Identification Erasmus MC University Medical Center Rotterdam Rotterdam the Netherlands; ^10^ Section of Chronobiology, Faculty of Health and Medical Sciences, University of Surrey Guildford UK

**Keywords:** diurnal rhythms, DNA methylation, epigenetics, immune response, sleep deprivation

## Abstract

Numerous hormones and genes exhibit diurnal 24‐hr rhythms that can also be affected by sleep deprivation. Here we studied diurnal rhythms in DNA methylation under a 24‐hr sleep/wake cycle and a subsequent 29 hr of continual wakefulness (1 night of sleep deprivation). Fifteen healthy men (19–35 years) spent 3 days/nights in a sleep laboratory: (1) adaptation; (2) baseline; (3) total sleep deprivation day/night. DNA methylation was analysed from peripheral blood leukocytes, collected every 3 hr for 45 hr (starting at 15:00 hours) during the baseline period and the total sleep deprivation period. Epigenome‐wide DNA methylation variation was assessed with the Infinium MethylationEPIC v2.0 Beadchip kit. Rhythm analysis was performed separately for the baseline and the total sleep deprivation time‐series data. Pairwise analysis between diurnal samples and sleep deprivation samples at the same timepoint was also carried out to detect differentially methylated positions related to sleep deprivation. Of all DNA methylation sites, 14% exhibited a diurnal rhythm in methylation on the baseline day/night that was altered by sleep deprivation. During sleep deprivation, the number of differentially methylated positions increased towards the end of the sleep deprivation period, with a dominating pattern of hypomethylation. Among differentially methylated positions, an enrichment of genes related to the FAS immune response pathway was detected. In conclusion, DNA methylation exhibits diurnal rhythmicity, and this time‐of‐day variation needs to be considered when studying DNA methylation as a biomarker in biomedical studies. In addition, the observed DNA methylation changes under wakefulness might serve as a mediator of sleep deprivation‐related immune response alterations.

## Graphical abstract

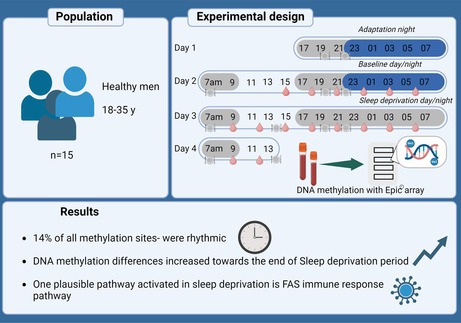

## 1 INTRODUCTION

DNA methylation (DNAm) is a chemical modification of DNA, and one of the key mechanisms that controls gene activity in different types of cells and tissues. There is a body of work exploring the relationship between DNAm and a variety of medical conditions, including cancer, and cardiovascular and neurological diseases (Yousefi et al., 2022). However, to date, this work has mostly utilized single timepoint sampling, and potential variation in DNAm across the 24‐hr day has not been considered.

Diurnal/daily 24‐hr rhythms have been demonstrated in most aspects of human physiology, including the metabolome, transcriptome and proteome (Mauvoisin & Gachon, 2020). Specifically, 8%–12% of the human transcriptome (Möller‐Levet et al., 2022) and 55%–65% of the human metabolome (Honma et al., 2020) in blood have been shown to be rhythmic under a light/dark, sleep/wake, feeding/fasting cycle (Chen et al., 2016). In addition, epigenetically variable cytosines have been shown to exhibit diurnal rhythms in mouse tissue and in human neutrophils extracted from single individuals (Oh et al., 2018; Oh et al., 2019). Furthermore, total sleep deprivation and insufficient/restricted sleep have been shown to affect human blood metabolite, protein and transcriptome rhythms (Depner et al., 2020; Honma et al., 2020; Weljie et al., 2015). Considering DNAm, in mouse cortex, using whole genome methylation sequencing, 0.35% of the possible differentially methylated positions (DMPs) were found to follow light/dark‐dependent rhythmic methylation patterns (Coulson et al., 2018) and, in mice, circadian clock‐controlled genes were regulated through methylation (Misra et al., 2023). Furthermore, in postmortem analysis of the human brain cortex, DNAm and histone acetylation were found to differ depending on the time of death, suggestive of some underlying diurnal rhythmicity (Lim et al., 2017). Here DNAm displayed an association with gene expression, where the nadir in DNAm preceded the gene expression peak by 1–3 hr (Lim et al., 2017).

Extended total sleep deprivation leads to severely hampered physiological and psychological functions, and it was demonstrated that after 72 hr of sleep deprivation individuals resemble the clinical state of acute psychosis or toxic delirium (Waters et al., 2018). The mechanisms involved in this transition are likely to include disruption in the brain glymphatic drainage (Lewis, 2021) and, at the molecular level, changes in DNAm and histone acetylation, and gene expression as observed in animal models (Gaine et al., 2018; Ventskovska et al., 2015). Previous studies on the effect of acute sleep deprivation on DNAm in humans have shown that 1 night of sleep deprivation significantly affects the DNAm profile (Nilsson et al., 2016), but the timeframe of development of these changes has not been explored. We previously observed a pattern of hypomethylation in blood leukocytes in patients with chronic sleep insufficiency (Lahtinen et al., 2019), while a study performed on a selected cohort of shift workers showed that the changes in DNAm relating to sleep insufficiency are dynamic (Lahtinen et al., 2021).

Depression and sleep deprivation are linked in terms of pathophysiology and response to treatment (Li et al., 2021). Clinically, DNAm has been linked to different forms of depression as well as to the antidepressant response. Finally, in idiopathic rapid eye movement (REM) sleep disorder, DNAm was shown to be predictive for phenoconversion (Li et al., 2021).

On the basis of the previous work, we hypothesized that DNAm would exhibit time of day variation and would also be affected by sleep deprivation. In the present study, we aimed to characterize diurnal rhythms in DNAm through analysis of time‐series samples collected sequentially for 45 hr across a 24‐hr day under controlled sleep/wake laboratory conditions, followed by a 21‐hr period of extended wakefulness.

## 2 MATERIALS AND METHODS

A sleep laboratory study was conducted at the University of Surrey. Full details of study eligibility, screening, the pre‐laboratory period and the study protocol have been previously published (Ackermann et al., 2012; Woelders et al., 2023). Briefly, the study participants comprised 15 healthy men aged 18–35 years with a mean age of 23.7 ± 5.4 years (mean ± SD). We focused on males to reduce any variability induced by the menstrual cycle and its associated cyclic hormonal activity as DNAm has been shown to be sensitive to both oestrogen and oestradiol (Boyne et al., 2017).

For 1 week prior to the experimental session, the participants' rest/activity rhythms were monitored by actigraphy and sleep diaries. Participants were then admitted to the laboratory for three consecutive days/nights in the sleep laboratory: adaptation night (day 1); the baseline day/night (day 2); and the sleep deprivation day/night (day 3). Breakfast, lunch, dinner and the snack were provided at the same clock time each day and were the same for all volunteers on all of the study days. Polysomnography recordings were obtained between 22:00 hours and 09:00 hours on nights 2 and 3 to assess sleep on night 2, and to confirm that the participants remained awake on night 3 (sleep deprivation).

**FIGURE 1:**
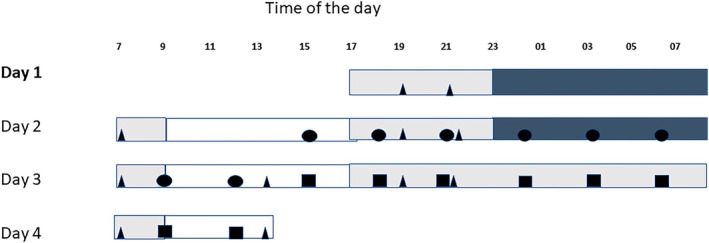
Study design. White bars indicate lights on (100 lux); light grey bars indicate dim light (< 5 lux) and dark grey bars indicate lights out (0 lux, sleep). Triangles indicate mealtimes. Blood samples were collected hourly. For this study, samples were analysed every 3 hr. Circles indicate blood sample collection during diurnal baseline study (15:00 hours–12:00 hours), and squares represent sampling during the wakefulness/sleep deprivation condition (15:00 hours–12:00 hours).

Blood samples for DNA analysis were collected in 1–2‐hr time intervals (Ackermann et al., 2012). In the present study, we examined the samples collected at 3‐hr intervals from 15:00 hours day 2 till 12:00 hours day 4, comprising the diurnal study (baseline) of 21 hours (15:00 hours day 2–12:00 hours day 3) and the total sleep deprivation night (15:00 hours day 3–12:00 hours day 4), altogether 16 samples per individual (Figure 1).

Sample collection and study design were performed according to the principles of the Declaration of Helsinki, with regard for Good Clinical Practice, and the study was given a favourable opinion by the University of Surrey Ethics Committees. All participants provided written informed consent prior to any study procedures being performed.

DNA was extracted at the Erasmus University Medical Center Rotterdam from blood leukocytes according to standard procedures. Samples were placed randomly on the plate, and individuals were anonymized and randomized in the sample list prior to the analysis. Batch and batch position were included as covariates in the statistical analyses of differentially methylated sites in the sleep deprivation analysis. We used a mixed linear model, where age, batch and batch position, probe and timepoint of sample were all used as random factors. Genome‐wide DNAm was measured by using the Infinium MethylationEPIC v2.0 Beadchip kit (llumina) targeting 864,935 Cytosine‐Guanine dinucleotide sites according to the manufacturer's protocol, corrected for background and followed by normalization with a subset quantile normalization approach (SWAN) (Houseman et al., 2012). The SWAN‐normalization process removes probes with detection *p*‐value > 0.01 (*N* = 9893), likewise probes with SNPs and cross‐reactive probes and probes in X and Y chromosomes. In total we removed 48,064 probes, which was 5.55% of the total probes in the array. Epigenetic clock effect was taken into consideration by using a Horvath Noob‐preprocess method. Data preprocessing and quality control (QC) was performed utilizing the R packages minfi (Aryee et al., 2014), minfiData (A. M. Hansen & Timp, 2022) and IlluminaHumanMethylationEPICmanifest (Hansen, 2016) using R software v3.6.1. After QC, 818,027 Cytosine‐Guanine dinucleotides were used for further analyses. Probes were annotated to nearest genes using previously described mapping to GRCH 38 HG19 according to previously described principles (Zhou et al., 2017) using the annotation function built into the R‐package “minfi”. To calculate *F*‐statistics, we used the R package “limma”, which takes into account paired sample designs where there are multiple measurements from the same source (Ritchie et al., 2015). Our goal was to evaluate global diurnal rhythmicity, also including “open sea” regions, and therefore we concentrated on Cytosine‐Guanine dinucleotide site level methylation change instead of larger regions.

## 3 DIURNAL RHYTHM ANALYSIS

To assess time of day differences in the degree of individual Cytosine‐Guanine dinucleotide site methylation between the baseline day/night and sleep deprivation conditions, rhythm analysis algorithms (see below) were employed to detect diurnal oscillating periods (~24‐hr rhythms) and acrophase (peak). The aforementioned Cytosine‐Guanine dinucleotide matrix, containing 818,027 Cytosine‐Guanine dinucleotide sites, every 3 hr across a 45‐hr time course for 15 participants, was split into two “halves” on the time axis. The first 24 hr (first nine samples) of the time course constituted the baseline diurnal day/night condition, and the last 24 hr (last nine samples) constituted the wakefulness/sleep deprivation condition. Whilst this resulted in a small overlap between the two time‐series, it was a necessary compromise to investigate a complete day cycle (24 hr) with the same number of samples under both conditions. The two data matrices were then independently subjected to *z*‐scoring prior to analysis by the R‐package “compareRhythms” (Pelikan et al., 2022) (v 1.0.1) utilizing the model selection method (“mod_sel”, <criterion = “aic”>, <schwarz_wt_cutoff = 0.6>, all other parameters set to default) to identify ~24‐hr diurnal rhythms in DNAm, and how these rhythms differed between the baseline and sleep deprivation conditions, i.e. “gain”, “loss”, “change” or “same” of rhythm between conditions or “arrhy” indicating no 24‐hr rhythm in either condition. The best model, i.e. “same”, “gain”, “loss”, “change”, “arrhythmic” to which Cytosine‐Guanine dinucleotide sites were assigned to was determined by the lowest Akaike information criterion (AIC) score. For a site to be successfully classified, the Akaike weight (relative likelihood) of the best model had to exceed the assigned “schwarz_wt_cutoff” threshold (≥ 0.6). This threshold is the conditional probability that the best fitting model (lowest AIC score) is also the true model (Pelikan et al., 2022). Cosinor analysis was employed to acquire acrophase data, using a *Z*‐scored data matric, and amplitude data, using the same data matrix prior to *Z*‐scoring, via the R package “DiscoRhythm” (Version 1.6.0; Carlucci et al., 2022) (parameters: <period = 24>, <method = “CS”>, <circular_t = FALSE>, unspecified parameters were left as default values) (Carlucci et al., 2022; Thaben & Westermark, 2014). A subset of rhythmic Cytosine‐Guanine dinucleotides was ordered by ascending acrophase (as determined by cosinor analysis) and then plotted as heatmaps to visualize and confirm the presence of ~24‐hr rhythms. Heatmaps were produced via MetaboAnalyst's R package (3.2.0) (Pang et al., 2020) with no additional filtering or data transformations, as data were previously *z*‐scored, except for group averaging data per timepoint. Further plots were produced using the R package ggplot2.

Diurnal rhythms in DNAm could potentially be influenced by the diurnal rhythms in white blood cell (WBC) abundance. To investigate this possibility, the data matrices were normalized to account for WBC rhythms, prior to being subjected to the previously described rhythm analysis (*Z*‐scoring, “compareRhythms”). The WBC counts required for this normalization were only available for 10 of the 15 participants; blood cell counts were independently measured across the time‐series for these 10 participants (Ackermann et al., 2012). Where a timepoint was missing (a total of three were missing across two participants), blood cell counts, per class, were imputed from the average of the two adjacent timepoints. Cell counts were available for 13 “classes” of WBCs as follows: total white bloods, granulocytes, lymphocytes, total T‐cells, total B‐cells, monocytes, total CD4 cells, CD4 memory, CD4 naïve, total CD8 cells, CD8 memory, CD8 naïve and natural killer/dendritic cells. Note that these categories are non‐exclusive, for example, “total CD8 cells count” is the sum of “CD8 memory” and “CD8 naïve” cells. Data were normalized by dividing the recorded DNAm‐value, for a given probe at a specific timepoint, by the number of WBCs per μl at the same timepoint. This process was iterated across all timepoints, across all probes, for the 10 aforementioned participants, and was independently repeated for the 13 categories of WBCs, producing 13 data matrices each normalized against a different blood cell population category. Post‐normalization, these 13 data matrices were subjected to *Z*‐scoring and rhythm analysis as detailed above. A further 14th data matrix was created consisting of the aforementioned 10 participants, with no normalization applied, but otherwise subjected to the same rhythm analysis method described above. The rhythm analysis performed therefore consisted of a non‐normalized dataset with the full cohort of 15 participants, presented in the main body of text; with a further 14 datasets (13 normalized against WBC populations) with the cohort of 10 participants presented within the supporting information (Table S2).

We monitored changes in DNAm on the sleep deprivation night (Figure 1; squares) as compared with the same timepoints on the previous sleep night (baseline night, circles; Figure 1). By analysis of this series of samples, we targeted the cumulative effect of sleep deprivation on DNAm, while the time‐of‐day influences were controlled by within‐subject comparison to samples collected at the same clock time on the previous baseline night.

The within‐subject comparison was performed by an empirical Bayes moderated *t*‐test to compare probe‐wise values from samples taken at the same clock times during and after sleep deprivation.

The significance of the effect of sleep deprivation on DNAm was tested using a variance ratio (*F*) test. Acquired *p*‐values were adjusted to control false discovery rate (FDR) multiple hypothesis testing using the Benjamini–Hochberg procedure. The beta coefficients from the tests were used to define a Cytosine‐Guanine dinucleotide site to be hyper‐ or hypomethylated.

If anticipating similar effect sizes to those we observed in our case–control study for insomnia and depression (Ämmälä et al., 2019; e.g. cg02263165 from the PPP25RC from the long term depression pathway), we had power of > 0.80 to detect deviation at a genome‐wide significance level of 5 × 10^−8^.

Pathway analysis was performed using the PANTHER pathway consisting of 177 primarily signalling pathways, as implemented in Enrichr (Chen E et al., 2023).

## 4 RESULTS

### 4.1 Diurnal rhythmicity

**FIGURE 2:**
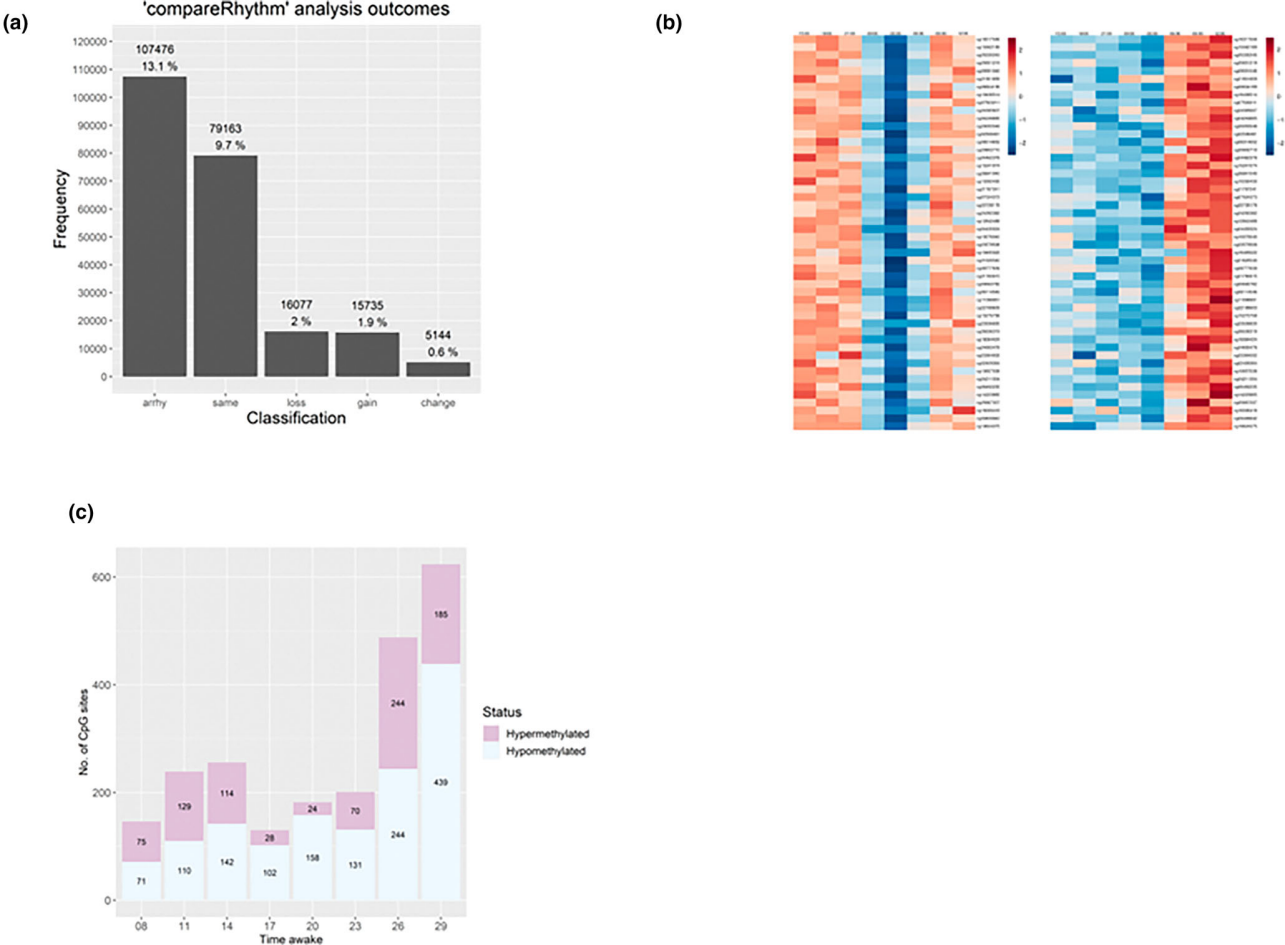
Diurnal rhythm analysis of DNA methylation (DNAm) and the effect of sleep deprivation on DNAm. (a) A frequency plot displaying the rhythmic classifications of CpG site DNAm as determined by “compareRhythms” model selection analysis. Classification categories: “arrhy” – non‐rhythmic, for ~24‐hr period, in both baseline sleep and sleep deprivation conditions; same – rhythmic for ~24‐hr period in both baseline sleep and sleep deprivation conditions; loss – rhythmic for ~24‐hr period in baseline sleep condition only; gain – rhythmic for ~24‐hr period in sleep deprivation condition only; change – rhythmic for ~24‐hr period in both baseline sleep and sleep deprivation conditions, but with a difference in phase between conditions. A total of 818,027 CpG sites were submitted for analysis of which 223,595 were classified, as shown in the figure, and 594,432 were unclassified. The percentage values displayed are relative to the classifiable sites as well as to full array of 818,027 CpG sites. (b) Heatmaps displaying *z*‐scored and group averaged changes in DNAm (red = high, blue = low) relative to time of day for CpG sites classified as “change” for the baseline sleep condition (left) and the sleep deprivation condition (right). Results shown are significant post‐false discovery rate (FDR) correction (*p* < 0.05, Benjamini–Hochberg). Sites classified as “change” were chosen to visually illustrate the change in acrophase determined by the “compareRhythms” analysis. Only the first 50 of 5144 of “change” classified CpG sites, as determined by ascending acrophase in the baseline sleep condition, are plotted here due to space limitations. Rows are matched between conditions such that row X in both heatmaps equates to the same CpG site. The time courses for the baseline sleep and sleep deprivation conditions start at 15:00 hours and end at 12:00 hours the next day. (c) The number of differentially methylated positions (DMPs) at *p* < 5 × 10^−4^ between sleep deprivation and baseline conditions. “Hypo” indicates that site is hypomethylated after sleep deprivation compared with baseline sleep night, and “Hyper” indicates that site is hypermethylated after sleep deprivation compared with baseline sleep night. Dashed line indicates expected number of DMPs in case of random appearance. Time awake is given in hours.

The time‐series DNAm data (every 3 hr across 45 hr) from 15 healthy males were subjected to 24‐hr rhythm analysis using cosinor analysis and “compareRhythms”, which incorporates validated rhythm analysis algorithms DODR (Detection Of Differential Rhythmicity) and RAIN (Rhythmicity Analysis Incorporating Non‐parametric methods; Carlucci et al., 2022; Pelikan et al., 2022; Thaben & Westermark, 2014; Thaben & Westermark, 2016). Of the 818,027 Cytosine‐Guanine dinucleotide (CpG) sites subjected to rhythm analysis, a total of 223,595 (27.3%) were successfully categorized as rhythmic (significant ~24‐hr oscillation in methylation) or non‐rhythmic (no significant ~24‐hr oscillation in methylation), post Benjamini–Hochberg FDR correction, whilst results on the remaining 594,432 were inconclusive. Of the total 223,595 Cytosine‐Guanine dinucleotide sites, 116,119 (14.2% of all sites) were classifiable as rhythmic (100,384 rhythmic at baseline and 100,042 rhythmic at sleep deprivation) and 107,476 (13.1% of all sites) as non‐rhythmic in the baseline or sleep deprivation conditions (Figure 2a; Table 1).

**TABLE 1:** Diurnal rhythmicity in baseline and in sleep deprivation conditions.

Classification	*N* of sites	% of classifiable sites (*N* = 223,595)	% of all sites in the array (*N* = 818,027)
Not classifiable	594,432	NA	72.7%
Arrhythmic	107,476	48.1%	13.1%
Same rhythmicity	79,163	35.4%	9.7%
Loss of rhythmicity	16,077	7.2%	2.0%
Gain of rhythmicity	15,735	7.0%	1.9%
Change in rhythmicity	5144	2.3%	0.6%

Of the Cytosine‐Guanine dinucleotide sites that exhibited 24‐hr rhythmic patterns (*n* = 116,119), the majority did so across both of these conditions (*n* = 79,163, 68.2% of rhythmic sites, classification “same”). An additional 5144 sites (4.4% of rhythmic sites) were rhythmic under both conditions, but with a change in acrophase (peak timing) both delayed and advanced (classification “change”). A subset of these “change” rhythmic Cytosine‐Guanine dinucleotide sites are displayed in Figure 2(b). In the baseline and sleep deprivation conditions, 16,077 (13.8% of rhythmic sites, classification “loss”) and 15,735 (13.6% of rhythmic sites, classification “gain”) Cytosine‐Guanine dinucleotide sites exhibited 24‐hr rhythmic patterns, respectively. A majority of 594,432 Cytosine‐Guanine dinucleotide sites (72.7% of total Cytosine‐Guanine dinucleotide sites) were not assigned a classification (“arrhythmic”, “same”, “change”, “gain” or “loss”) as they failed to pass the assigned AIC, an estimator of prediction error in our data, threshold (≥ 0.6) within the rhythm analysis.

Of the Cytosine‐Guanine dinucleotide sites with rhythmic DNAm in both baseline and sleep deprivation conditions (*n* = 84,307, classification “same” + “change”), ~87.2% (*n* = 73,520) possessed an acrophase, as determined by independent cosinor analysis, ± 3 hr from sleep onset (20:00 hours–02:00 hours). However, for these same sites at the same clock time in the sleep deprivation condition, only ~40.7% (*n* = 34,349) peaked at this time. The measured acrophases and thus “peak” methylation for the majority of Cytosine‐Guanine dinucleotide sites in the sleep deprivation condition occurred earlier in the evening, with ~77.8% (*n* = 65,665) peaking between 18:00 hours and 23:00 hours. Only a small proportion of Cytosine‐Guanine dinucleotide sites (*n* = 5144, 4.4%) were classified as differing in acrophase between the baseline and sleep deprivation conditions (“change”).

Because diurnal rhythms in DNAm could potentially be influenced by diurnal rhythms in WBC composition, the DNAm data matrices in a subset of participants (*n* = 10) were individually normalized by dividing their DNAm‐value at each timepoint by the number of blood cells per μl at the same timepoint (*n* = 13 cell types). Post‐normalization, these 13 data matrices were also subjected to *Z*‐scoring and rhythm analysis as before. With most cell populations (*n* = 9), rhythmicity typically increased after normalization in both baseline and sleep deprivation conditions (Table S2). This is probably due to reduced variance, as the underlying blood cell rhythms that may have been enforcing/masking rhythms in CpG methylation have been removed. By contrast, normalization to CD4 cells, monocytes, B‐cells and natural killer/dendritic cells resulted in reduced rhythmicity.

### 4.2 Sleep deprivation

To assess the effect of total sleep deprivation, the data were compared in a pairwise manner (samples from the same time of day baseline versus sleep deprivation). None of the pairwise comparisons revealed statistically significant changes in DNAm induced by sleep deprivation after correction for multiple testing (Table S1). However, the number of DMPs increased at the last two sampling timepoints, after 26 hr and 29 hr of wakefulness compared with the baseline day. For these two timepoints, there were 487 and 624 DMPs, respectively (Table 2). Of these 624 sites, only a minority were classified as rhythmic (Table S3). The number of DMPs with uncorrected *p*‐value of 10^−4^ significantly increased after 29 hr of wakefulness (*n* = 55,138). Interestingly, after 14 hr of wakefulness (21:00 hours day 3), there was a peak of DMPs, with 5 × 10^−4^ uncorrected *p*‐values. However, at that timepoint, the number of DMPs at *p* < 5.0 × 10^−4^ remained nearly twofold lower compared with after 29 hr wakefulness. At the two last timepoints of 26 hr and 29 hr of wakefulness, both the number of hypo‐ and hypermethylated DMPs increased, but there was an excess of hypomethylated DMPs, 70% of sites (439/624) were hypomethylated at *p* < 5.0 × 10^−4^ after 29 hr of wakefulness (the last timepoint of sleep deprivation; Figure 2c).

**TABLE 2:** DMPs at 8–29 hr since time awake, as compared with the equivalent diurnal baseline timepoint 24 hr earlier (accumulating numbers) under a sleep/wake cycle.

Timepoint (T1)^a^	Timepoint (T2)^a^	Time awake^b^	*N*(DMPs) with *p* < **						
			5 × 10^−8^	5 × 10^−7^	5 × 10^−6^	5 × 10^−5^	5 × 10^−4^	5 × 10^−3^	5 × 10^−2^
D2 15:00 hours	D3 15:00 hours	8 hr	0	1	1	10	146	1639	19,549
D2 18:00 hours	D3 18:00 hours	11 hr	0	0	2	19	239	2286	25,090
D2 21:00 hours	D3 21:00 hours	14 hr	0	0	1	19	256	3226	57,536
D2 24:00 hours	D3 24:00 hours	17 hr	0	1	1	19	130	1431	18,288
D3 03:00 hours	D4 03:00 hours	20 hr	0	0	1	27	182	2130	24,469
D3 06:00 hours (19)	D4 06:00 hours (43)	23 hr	0	1	3	21	201	1978	23,156
D3 09:00 hours (22)	D4 09:00 hours (46)	26 hr	0	1	10	57	487	4012	34,853
D3 12:00 hours (25)	D4 12:00 hours (49)	29 hr	0	0	8	62	624	5619	55,138

^a^ (T1) Reference timepoint: diurnal baseline; (T2) sleep deprivation timepoint.

^b^ Wake‐time was at 07:00 hours on D2, lights went out at 23:00 hours. DMP, differentially methylated position.

### 4.3 Pathway analyses

Given the results from the pairwise comparison, we focused on pathway analysis on the DMPs from the last timepoint after 29 hr wakefulness (sleep deprivation), collected on day 4 at 12:00 hours (Figure 2c). To this end, we selected the top 1000 DMPs, annotated into 755 unique genes, for pathway analysis. This allowed a manageable size of DMPs for secondary analyses yet providing a clear pattern of methylation change in DMPs towards the end of sleep deprivation. Because sleep deprivation can affect WBC counts (Ackermann et al., 2012), and WBC can affect DNAm (Houseman et al., 2012), we aimed to ensure that alterations in site‐specific methylation following sleep deprivation were not solely due to changes in WBC composition. To investigate, we assessed the correlation between the relative proportions of 13 different WBC types and the methylation status of each Cytosine‐Guanine dinucleotide site at the final (T49) timepoint of sleep deprivation. Our hypothesis was that if methylation primarily aligns with WBC composition, there would be a stronger correlation between methylation and WBC composition than expected by chance. Using an alpha error rate of 0.05, running 13 correlation analyses 1000 times would yield 650 significant correlations. In our analysis (Table S1), we found a significant correlation in 1073 cases, slightly exceeding the expected value. After implementing the FDR‐correction, none of the correlations retained significance at the adjusted 0.05 level (Table S1).

Pathway analysis with Panther in Enrichr resulted in the following top pathways: FAS signalling pathway related to programmed cell death (*p*_uncorrectedl_ = 7.0 × 10^−4^; *p*_corrected_ = 5.7 × 10^−2^), T cell activation (*p*_uncorr_ = 4.45 × 10^−3^; *p*_corr._ = 0.180) and Alzheimer's disease‐amyloid secretase pathway (*p*_uncorr_ = 1.46 × 10^−2^; *p*_corr_. = 0.261). Genes included in the FAS pathway and their respective methylation changes during sleep deprivation are presented in Table 3. There were six genes in the FAS pathway that were represented in the DMPs list after sleep deprivation: *MAPK10*, *DFFB*, *JUN*, *SCIN*, *PARP4* and *LMNA*, the latter with two separate Cytosine‐Guanine dinucleotides (Table 3).

**TABLE 3:** Genes and DMPs in FAS signalling pathway.

Gene	Probe	Relation to regulatory position	HYPO/HYPER after SD	Change towards	*p*‐Value
*SCIN*	cg11040906	Body	Hypo	Hypo	5.8E^−5^
*LMNA*	cg26307728	N_Shelf	Hypo	Hypo	3.3E^−4^
*LMNA*	cg15644472	NA	Hypo	Hypo	3.7E^−4^
*PARP4*	cg04120842	Body	Hypo	Hypo	4.4E^−4^
*JUN*	cg03256465	Island	Hypo	Hypo	4.6E^−4^
*MAPK10*	cg22223941	TSS1500	Hyper	Hyper	6.0E^−4^
*DFFB*	cg12744321	Island	Hypo	Hypo	7.1E^−4^

Change towards, direction of change during sleep deprivation; *p*‐value, uncorrected *p*‐value between diurnal baseline night measurement and after sleep deprivation; SD, sleep deprivation.

## 5 DISCUSSION

To our knowledge, this is the first study in humans exploring the timeframe in which DNAm changes across a 24‐hr day/night period, and how DNAm responds to a single night of total sleep deprivation. We detected clear diurnal rhythmicity in approximately half the classifiable DNAm sites. We also found a non‐linear increase of DMPs towards the end of the sleep deprivation period, suggesting that methylation increases with the duration of sleep deprivation. The largest change was observed between 23 hr and 29 hr of wakefulness, with nearly a threefold increase of DMPs during the last 6 hr of sleep deprivation. Our last sampling timepoint occurred after 29 hr of continual wakefulness; therefore, we do not know if this observed increase in DMPs would have continued with an extended sleep deprivation period. Our findings show that sleep deprivation enhanced the existing methylation status, for example, sites hypomethylated at the beginning of the experiment were even more hypomethylated after sleep deprivation and hypermethylated sites were even more hypermethylated after sleep deprivation.

We found that over half of all the DNAm sites that could be classified for rhythmicity were indeed statistically significantly rhythmic (116,119 of 223,595 Cytosine‐Guanine dinucleotide sites, 51.9%). This finding is important as DNAm is being used as a biomarker and thus the effects of time of day of sampling should be more carefully considered in future studies. Furthermore, we showed that sleep deprivation had a differential effect on this rhythmicity as the acrophase of numerous Cytosine‐Guanine dinucleotide sites either peaked later or earlier in the sleep deprivation condition compared with the sleep condition or did not change at all (Figure S1). It is well documented that either lack of sleep or mistimed sleep perturbs diurnal rhythms either by shifting their phase or dampening their amplitude. From our data it appears that DNAm is no exception to this generalization (Potter et al., 2016).

A shift in the acrophase of DNAm rhythms was observed for a number of Cytosine‐Guanine dinucleotide sites between the baseline and sleep deprivation conditions. The change in acrophase between these two conditions is likely indicative of sleep and sleep deprivation being important behavioural cues in the regulation of DNAm as seen for these Cytosine‐Guanine dinucleotide sites. A similar conclusion was drawn with reference to diurnal metabolite rhythms and total sleep deprivation in a previous metabolomics analysis of samples from this cohort (Davies et al., 2014). Moreover, in a primate model, the diurnal rhythms of DNA transcription also appeared organized around the sleep/wake cycle, with a “transcriptionally quiescent period” observed during sleep and the majority of observed transcripts peaking during wakefulness across several different tissues (Mure et al., 2018).

Notably, our study did not reveal a unidirectional “loss” of rhythmicity that one might expect as a result of sleep deprivation. On the contrary, during sleep deprivation we found a “switch” in rhythmic DNAm for a subset of Cytosine‐Guanine dinucleotide sites (*n* = 16,077 losing rhythmicity, *n* = 15,735 gaining rhythmicity). This switch in DNAm may reflect the body's stress response to sleep deprivation, which has variable and wide‐ranging consequences, such as previously noted alterations in metabolism or cognition in response to sleep deprivation (Kayser et al., 2022). Our results not only identify clear diurnal DNAm rhythms in healthy males but also suggest a more complex impact of sleep deprivation on diurnal DNAm rhythms than simply loss/dampening of the rhythm.

There are numerous factors that may contribute to the observed daily rhythms in DNAm (e.g. light/dark cycle; feeding/fasting; sleep/wake) as well as changes in WBC composition across the 24‐hr day. Correcting the DNAm data matrices for 13 WBC populations did not significantly affect the diurnal DNAm rhythmicity in the baseline and sleep deprivation conditions. Likewise, correlation analysis between methylation and WBC composition at the last sampling timepoint of sleep deprivation (29 hr of wakefulness) did not show any significant relationship.

Our finding of extended sleep deprivation associating with increased DNA hypomethylation is somewhat contrary to previous findings where acute sleep deprivation has been linked with hypermethylation at the end of 24‐hr sleep deprivation period (Skuladottir et al., 2016). However, our results show that changes in methylation status occur in the late part of sleep deprivation (after 26 hr and 29 hr of sleep deprivation). It is therefore possible that the hypomethylation we detected in our study is a late‐stage response to sleep deprivation compared with hypermethylation. Our findings are in line with previous findings of Lahtinen and colleagues (Lahtinen et al., 2019), who showed an increase of hypomethylation over hypermethylation in response to chronic sleep insufficiency. Furthermore, another study by Nilsson and colleagues (Nilsson et al., 2016) found that sleep deprivation in healthy men invoked hypomethylation of the *ING5* gene as a response, and this hypomethylation was correlated with gene expression (Nilsson et al., 2016).

Due to the small sample size, we could not detect genome‐wide significant effects after FDR correction of sleep deprivation on methylation. However, to explore plausible pathways affected by sleep deprivation, we proceeded to pathway analysis with the most prominent sites according to the uncorrected *p*‐values. After 29 hr of wakefulness, our gene enrichment analysis highlighted genes related to a pathway modulating inflammatory responses, the FAS signalling pathway. The FAS pathway is a chemokine‐driven pathway directing the traffic and migration of immune cells in tissues as well as recruiting new immune cells to participate in the immune response. Here, we found DMPs influenced by sleep deprivation in six genes in that pathway (*SCIN*, *LMNA*, *PARP4*, *JUN*, *MAPK10*, *DFFB*). There is evidence that methylation alters gene expression for some FAS‐pathway‐related genes. Methylation alterations of SCIN and PARP4 have been associated with RNA expression (Weixler et al., 2021; Zhang et al., 2018), in a way that hypomethylation is associated with increased gene expression. Little is known, however, about the exact impact of a single Cytosine‐Guanine dinucleotide's effect on gene expression. Of sites in the FAS‐pathway, only the *JUN* associated site seems to have a clear connection to expression (Hao et al., 2021).

Animal studies have shown that acute sleep deprivation can differentially alter the immune system compared with chronic sleep deprivation. In one study, acute sleep deprivation decreased IL1β, but chronic sleep deprivation did not have such an effect, whereas TNFα did not change in acute sleep deprivation but increased in chronic sleep deprivation (Fahmawi et al., 2023). Thus, it is likely that sleep deprivation disrupts immune function in several ways depending on its duration and whether it is acute or chronic sleep deprivation. Insomnia symptoms have been previously associated with advanced epigenetic aging and immune senescence (Carroll et al., 2017), but no specific DMPs were studied in this relatively large sample of midwives, neither was the genetic risk for insomnia considered.

The strength of our study, next to covering a large number of DNAm sites across the epigenome, includes strict control of the confounding factors that may affect diurnal rhythms as well as relatively good sampling resolution (every 3 hr across 45 hr). All study participants went to bed at the same time, ate the same food and lived in the same light/dark cycle in a highly controlled laboratory study. Also, the sleep deprivation period was comprised of 29 hr of controlled wakefulness allowing us to detect the effects of longer sleep deprivation on DNAm. All participants were males, which eliminates menstrual cycle effects, but at the same time makes generalizing the results more difficult. DNAm has been shown to be sensitive to both oestrogen and oestradiol (Boyne et al., 2017), and thus it is a source of possible confounding that we wanted to eliminate in this first study investigating the effect of sleep deprivation and time of day on DNAm. Moreover, males are better suited to high‐resolution blood sampling (every hour for 48 hr) with less incidence of vein collapse. The DNAm analysis was performed on time‐series samples obtained from males undertaking a sleep deprivation study (Ackermann et al., 2012; Davies et al., 2014), no females were studied. A weakness of our study is that the number of individuals studied was relatively small (*N* = 15). Although for controlled sleep and circadian laboratory ‐omics studies, this sample size is common, it may be too small to detect genome‐wide findings. In addition, we focused on methylation and were not able to analyse hydroxy methylation and the changes therein. It is possible that hydroxy methylation could act differentially from methylation as shown in a study where acute sleep deprivation increased global methylation but decreased hydroxy methylation.

In conclusion, we demonstrate that DNAm varies across time of day. Clear diurnal rhythms in DNAm were identified, which were altered differentially by sleep deprivation, with most rhythms staying the same, some being lost, some being gained and few showing altered peak times in sleep deprivation. We also observed a non‐linear increase in DNAm towards the end of sleep deprivation, which was directed primarily towards hypomethylation. The FAS signalling pathway highlighted by our analysis may react to sleep deprivation by hypomethylation, which could be one explanatory mechanism underlying the connection between sleep deprivation and altered immune responses. Our findings suggest that the length of the sleep deprivation period is an important factor to consider in future sleep deprivation research as it may be that some effects are only taking place after a long period of sleep deprivation. Overall, the effect of time of day and diurnal DNAm rhythms should be considered when using DNAm as a biomarker in future medical and other studies, especially when studying the effects of sleep deprivation, as sleep deprivation can change DNAm rhythmicity in a complex manner.

## AUTHOR CONTRIBUTIONS

**Antti‐Jussi Ämmälä:** Writing – original draft; writing – review and editing; formal analysis. **Thomas P. M. Hancox:** Writing – original draft; writing – review and editing; software; formal analysis; data curation. **Fan Qiuyu:** Writing – original draft; software; formal analysis. **Alexandra Lahtinen:** Methodology; software; writing – review and editing. **Sonja Sulkava:** Conceptualization; methodology; writing – review and editing. **Victoria L. Revell:** Conceptualization; writing – review and editing; investigation; methodology. **Katrin Ackermann:** Conceptualization; investigation; methodology; writing – review and editing. **Manfred Kayser:** Conceptualization; investigation; methodology; writing – review and editing. **Debra J. Skene:** Conceptualization; investigation; funding acquisition; methodology; supervision; writing – review and editing; writing – original draft; resources. **Tiina Paunio:** Conceptualization; investigation; funding acquisition; methodology; writing – review and editing; supervision; resources.

## CONFLICT OF INTEREST STATEMENT

Financial Disclosure: none. Non‐financial Disclosure: none.

## Supporting information


**FIGURE S1.** A scatter plot of the Baseline acrophase versus Sleep Deprivation acrophase per rhythmic CpG site. Comparison of the acrophase (peak time) of DNAme in sleep versus sleep deprivation conditions per rhythmic CpG site. Points on the thin black line exhibit the same acrophase between conditions.

**TABLE S1.** Correlation between the relative proportions of 13 different white blood cell types and the methylation status of each Cytosine‐Guanine dinucleotide site at the final (T49) timepoint of sleep deprivation FDR, false discovery rate.

**TABLE S2.** The number of CpG sites, of the 223,595 classified in the original analysis (Figure 2a), that exhibit 24‐hr rhythms post‐normalization to account for 24‐hr rhythms in white blood cell composition.

**TABLE S3.** CpG sites in the sleep deprivation condition that differed from baseline and were also classified as rhythmic Following sleep deprivation, we identified 624 sites that differed from baseline with an uncorrected *p*‐value of 10^−4^. Of these, 150 were classified as rhythmic using “compareRhythm” analysis.

## Data Availability

The data that support the findings of this study are openly available in Daily rythm in DNA methylation at https://osf.io/qx7w3/, reference number DOI 10.17605/OSF.IO/QX7W3.

## References

[jsr14438-bib-0001] A. M. Hansen, K. D. , & Timp, W. (2022). Minfi: A flexible and comprehensive Bioconductor package for the analysis of Infinium DNA Methylation microarrays. 10.1093/bioinformatics/btu049 PMC4016708 24478339

[jsr14438-bib-0002] Ackermann, K. , Revell, V. L. , Lao, O. , Rombouts, E. J. , Skene, D. J. , & Kayser, M. (2012). Diurnal rhythms in blood cell populations and the effect of acute sleep deprivation in healthy young men. Sleep, 35, 933–940. 22754039 10.5665/sleep.1954 PMC3369228

[jsr14438-bib-0003] Ämmälä, A. J. , Urrila, A. S. , Lahtinen, A. , Santangeli, O. , Hakkarainen, A. , Kantojärvi, K. , Castaneda, A. E. , Lundbom, N. , Marttunen, M. , & Paunio, T. (2019). Epigenetic dysregulation of genes related to synaptic long‐term depression among adolescents with depressive disorder and sleep symptoms. Sleep Medicine, 61, 95–103. 31395523 10.1016/j.sleep.2019.01.050

[jsr14438-bib-0004] Aryee, M. J. , Jaffe, A. E. , Corrada‐Bravo, H. , Ladd‐Acosta, C. , Feinberg, A. P. , Hansen, K. D. , & Irizarry, R. A. (2014). Minfi: A flexible and comprehensive Bioconductor package for the analysis of Infinium DNA methylation microarrays. Bioinformatics, 30, 1363–1369. 24478339 10.1093/bioinformatics/btu049 PMC4016708

[jsr14438-bib-0005] Boyne, D. J. , Friedenreich, C. M. , McIntyre, J. B. , Stanczyk, F. Z. , Courneya, K. S. , & King, W. D. (2017). Endogenous sex hormone exposure and repetitive element DNA methylation in healthy postmenopausal women. Cancer Causes & Control, 28, 1369–1379. 28929436 10.1007/s10552-017-0958-z

[jsr14438-bib-0006] Carlucci, M. , Kriščiūnas, A. , Li, H. , Gibas, P. , Koncevičius, K. , Petronis, A. , & Oh, G. (2022). DiscoRhythm: An easy‐to‐use web application and R package for discovering rhythmicity. Bioinformatics, 38, 882. 34874990 10.1093/bioinformatics/btab517 PMC8756186

[jsr14438-bib-0007] Carroll, J. E. , Irwin, M. R. , Levine, M. , Seeman, T. E. , Absher, D. , Assimes, T. , & Horvath, S. (2017). Epigenetic aging and immune senescence in women with insomnia symptoms: Findings from the Women's Health Initiative study. Biological Psychiatry, 81, 136–144. 27702440 10.1016/j.biopsych.2016.07.008 PMC5536960

[jsr14438-bib-0008] Chen, C. Y. , Logan, R. W. , Ma, T. , Lewis, D. A. , Tseng, G. C. , Sibille, E. , & McClung, C. A. (2016). Effects of aging on circadian patterns of gene expression in the human prefrontal cortex. Proceedings of the National Academy of Sciences of the United States of America, 113, 206–211. 26699485 10.1073/pnas.1508249112 PMC4711850

[jsr14438-bib-0009] Chen E, E. J. R. , Lachmann, A. , et al. (2023). Enrichr. Icahn School of Medicine at Mount Sinai. p Webtool.

[jsr14438-bib-0010] Coulson, R. L. , Yasui, D. H. , Dunaway, K. W. , Laufer, B. I. , Vogel Ciernia, A. , Zhu, Y. , Mordaunt, C. E. , Totah, T. S. , & LaSalle, J. M. (2018). Snord116‐dependent diurnal rhythm of DNA methylation in mouse cortex. Nature Communications, 9, 1616. 10.1038/s41467-018-03676-0 PMC5915486 29691382

[jsr14438-bib-0011] Davies, S. K. , Ang, J. E. , Revell, V. L. , Holmes, B. , Mann, A. , Robertson, F. P. , Cui, N. , Middleton, B. , Ackermann, K. , Kayser, M. , Thumser, A. E. , Raynaud, F. I. , & Skene, D. J. (2014). Effect of sleep deprivation on the human metabolome. Proceedings of the National Academy of Sciences of the United States of America, 111, 10761–10766. 25002497 10.1073/pnas.1402663111 PMC4115565

[jsr14438-bib-0012] Depner, C. M. , Cogswell, D. T. , Bisesi, P. J. , Markwald, R. R. , Cruickshank‐Quinn, C. , Quinn, K. , Melanson, E. L. , Reisdorph, N. , & Wright, K. P., Jr. (2020). Developing preliminary blood metabolomics‐based biomarkers of insufficient sleep in humans. Sleep, 43, 1–13. 10.1093/sleep/zsz321 PMC7355401 31894238

[jsr14438-bib-0013] Fahmawi, A. , Khalifeh, M. , Alzoubi, K. H. , & Rababa'h, A. M. (2023). The effects of acute and chronic sleep deprivation on the immune profile in the rat. Current Molecular Pharmacology, 16(1), 101–108. 10.2174/1874467215666220316104321 35297357

[jsr14438-bib-0014] Gaine, M. E. , Chatterjee, S. , & Abel, T. (2018). Sleep deprivation and the epigenome. Frontiers in Neural Circuits, 12, 14. 29535611 10.3389/fncir.2018.00014 PMC5835037

[jsr14438-bib-0015] Hansen, K. D. (2016). IlluminaHumanMethylationEPICmanifest: Manifest for Illumina's EPIC methylation arrays. R Package Version 0.3.0.

[jsr14438-bib-0016] Hao, Y. , Hao, S. , Andersen‐Nissen, E. , Mauck, W. M., III , Zheng, S. , Butler, A. , Lee, M. J. , Wilk, A. J. , Darby, C. , Zager, M. , Hoffman, P. , Stoeckius, M. , Papalexi, E. , Mimitou, E. P. , Jain, J. , Srivastava, A. , Stuart, T. , Fleming, L. M. , Yeung, B. , … Satija, R. (2021). Integrated analysis of multimodal single‐cell data. Cell, 184, 3573–3587.e3529. 34062119 10.1016/j.cell.2021.04.048 PMC8238499

[jsr14438-bib-0017] Honma, A. , Revell, V. L. , Gunn, P. J. , Davies, S. K. , Middleton, B. , Raynaud, F. I. , & Skene, D. J. (2020). Effect of acute total sleep deprivation on plasma melatonin, cortisol and metabolite rhythms in females. The European Journal of Neuroscience, 51, 366–378. 30929284 10.1111/ejn.14411 PMC7027445

[jsr14438-bib-0018] Houseman, E. A. , Accomando, W. P. , Koestler, D. C. , Christensen, B. C. , Marsit, C. J. , Nelson, H. H. , Wiencke, J. K. , & Kelsey, K. T. (2012). DNA methylation arrays as surrogate measures of cell mixture distribution. BMC Bioinformatics, 13, 86. 22568884 10.1186/1471-2105-13-86 PMC3532182

[jsr14438-bib-0019] Kayser, K. C. , Puig, V. A. , & Estepp, J. R. (2022). Predicting and mitigating fatigue effects due to sleep deprivation: A review. Frontiers in Neuroscience, 16, 930280. 35992930 10.3389/fnins.2022.930280 PMC9389006

[jsr14438-bib-0020] Lahtinen, A. , Häkkinen, A. , Puttonen, S. , Vanttola, P. , Viitasalo, K. , Porkka‐Heiskanen, T. , Härmä, M. , & Paunio, T. (2021). Differential DNA methylation in recovery from shift work disorder. Scientific Reports, 11, 2895. 33536559 10.1038/s41598-021-82627-0 PMC7858604

[jsr14438-bib-0021] Lahtinen, A. , Puttonen, S. , Vanttola, P. , Viitasalo, K. , Sulkava, S. , Pervjakova, N. , Joensuu, A. , Salo, P. , Toivola, A. , Härmä, M. , Milani, L. , Perola, M. , & Paunio, T. (2019). A distinctive DNA methylation pattern in insufficient sleep. Scientific Reports, 9, 1193. 30718923 10.1038/s41598-018-38009-0 PMC6362278

[jsr14438-bib-0022] Lewis, L. D. (2021). The interconnected causes and consequences of sleep in the brain. Science, 374, 564–568. 34709917 10.1126/science.abi8375 PMC8815779

[jsr14438-bib-0023] Li, Y. , Hao, S. , Zhang, H. , Mao, W. , Xue, J. , Zhang, Y. , Cai, Y. , & Chan, P. (2021). Hypomethylation of SNCA in idiopathic REM sleep behavior disorder associated with phenoconversion. Movement Disorders, 36, 955–962. 33340152 10.1002/mds.28421

[jsr14438-bib-0024] Lim, A. S. , Klein, H.‐U. , Yu, L. , Chibnik, L. B. , Ali, S. , Xu, J. , Bennett, D. A. , & De Jager, P. L. (2017). Diurnal and seasonal molecular rhythms in human neocortex and their relation to Alzheimer's disease. Nature Communications, 8, 14931. 10.1038/ncomms14931 PMC5382268 28368004

[jsr14438-bib-0025] Mauvoisin, D. , & Gachon, F. (2020). Proteomics in circadian biology. Journal of Molecular Biology, 432, 3565–3577. 31843517 10.1016/j.jmb.2019.12.004

[jsr14438-bib-0026] Misra, N. , Damara, M. , Ye, T. , & Chambon, P. (2023). The circadian demethylation of a unique intronic deoxymethylCpG‐rich Island boosts the transcription of its cognate circadian clock output gene. Proceedings of the National Academy of Sciences of the United States of America, 120, e2214062120. 36791105 10.1073/pnas.2214062120 PMC9974474

[jsr14438-bib-0027] Möller‐Levet, C. S. , Laing, E. E. , Archer, S. N. , & Dijk, D. J. (2022). Diurnal and circadian rhythmicity of the human blood transcriptome overlaps with organ‐ and tissue‐specific expression of a non‐human primate. BMC Biology, 20, 63. 35264172 10.1186/s12915-022-01258-7 PMC8905855

[jsr14438-bib-0028] Mure, L. S. , le, H. D. , Benegiamo, G. , Chang, M. W. , Rios, L. , Jillani, N. , Ngotho, M. , Kariuki, T. , Dkhissi‐Benyahya, O. , Cooper, H. M. , & Panda, S. (2018). Diurnal transcriptome atlas of a primate across major neural and peripheral tissues. Science, 359. 10.1126/science.aao0318 PMC5924732 29439024

[jsr14438-bib-0029] Nilsson, E. K. , Boström, A. E. , Mwinyi, J. , & Schiöth, H. B. (2016). Epigenomics of total acute sleep deprivation in relation to genome‐wide DNA methylation profiles and RNA expression. Omics, 20, 334–342. 27310475 10.1089/omi.2016.0041 PMC4926204

[jsr14438-bib-0030] Oh, G. , Ebrahimi, S. , Carlucci, M. , Zhang, A. , Nair, A. , Groot, D. E. , Labrie, V. , Jia, P. , Oh, E. S. , Jeremian, R. H. , Susic, M. , Shrestha, T. C. , Ralph, M. R. , Gordevičius, J. , Koncevičius, K. , & Petronis, A. (2018). Cytosine modifications exhibit circadian oscillations that are involved in epigenetic diversity and aging. Nature Communications, 9, 644. 10.1038/s41467-018-03073-7 PMC5811577 29440637

[jsr14438-bib-0031] Oh, G. , Koncevičius, K. , Ebrahimi, S. , Carlucci, M. , Groot, D. E. , Nair, A. , Zhang, A. , Kriščiūnas, A. , Oh, E. S. , Labrie, V. , Wong, A. H. C. , Gordevičius, J. , Jia, P. , Susic, M. , & Petronis, A. (2019). Circadian oscillations of cytosine modification in humans contribute to epigenetic variability, aging, and complex disease. Genome Biology, 20, 2. 30606238 10.1186/s13059-018-1608-9 PMC6317262

[jsr14438-bib-0032] Pang, Z. , Chong, J. , Li, S. , & Xia, J. (2020). MetaboAnalystR 3.0: Toward an optimized workflow for global metabolomics. Metabolites, 10. 10.3390/metabo10050186 PMC7281575 32392884

[jsr14438-bib-0033] Pelikan, A. , Herzel, H. , Kramer, A. , & Ananthasubramaniam, B. (2022). Venn diagram analysis overestimates the extent of circadian rhythm reprogramming. The FEBS Journal, 289, 6605–6621. 34189845 10.1111/febs.16095

[jsr14438-bib-0034] Potter, G. D. , Skene, D. J. , Arendt, J. , Cade, J. E. , Grant, P. J. , & Hardie, L. J. (2016). Circadian rhythm and sleep disruption: Causes, metabolic consequences, and countermeasures. Endocrine Reviews, 37, 584–608. 27763782 10.1210/er.2016-1083 PMC5142605

[jsr14438-bib-0035] Ritchie, M. E. , Phipson, B. , Wu, D. , Hu, Y. , Law, C. W. , Shi, W. , & Smyth, G. K. (2015). Limma powers differential expression analyses for RNA‐sequencing and microarray studies. Nucleic Acids Research, 43, e47. 25605792 10.1093/nar/gkv007 PMC4402510

[jsr14438-bib-0036] Skuladottir, G. V. , Nilsson, E. K. , Mwinyi, J. , & Schiöth, H. B. (2016). One‐night sleep deprivation induces changes in the DNA methylation and serum activity indices of stearoyl‐CoA desaturase in young healthy men. Lipids in Health and Disease, 15, 137. 27562731 10.1186/s12944-016-0309-1 PMC5000434

[jsr14438-bib-0037] Thaben, P. F. , & Westermark, P. O. (2014). Detecting rhythms in time series with RAIN. Journal of Biological Rhythms, 29, 391–400. 25326247 10.1177/0748730414553029 PMC4266694

[jsr14438-bib-0038] Thaben, P. F. , & Westermark, P. O. (2016). Differential rhythmicity: Detecting altered rhythmicity in biological data. Bioinformatics, 32, 2800–2808. 27207944 10.1093/bioinformatics/btw309

[jsr14438-bib-0039] Ventskovska, O. , Porkka‐Heiskanen, T. , & Karpova, N. N. (2015). Spontaneous sleep‐wake cycle and sleep deprivation differently induce Bdnf1, Bdnf4 and Bdnf9a DNA methylation and transcripts levels in the basal forebrain and frontal cortex in rats. Journal of Sleep Research, 24, 124–130. 25223586 10.1111/jsr.12242

[jsr14438-bib-0040] Waters, F. , Chiu, V. , Atkinson, A. , & Blom, J. D. (2018). Severe sleep deprivation causes hallucinations and a gradual progression toward psychosis with increasing time awake. Frontiers in Psychiatry, 9, 303. 30042701 10.3389/fpsyt.2018.00303 PMC6048360

[jsr14438-bib-0041] Weixler, L. , Schäringer, K. , Momoh, J. , Lüscher, B. , Feijs, K. L. H. , & Žaja, R. (2021). ADP‐ribosylation of RNA and DNA: From in vitro characterization to in vivo function. Nucleic Acids Research, 49, 3634–3650. 33693930 10.1093/nar/gkab136 PMC8053099

[jsr14438-bib-0042] Weljie, A. M. , Meerlo, P. , Goel, N. , Sengupta, A. , Kayser, M. S. , Abel, T. , Birnbaum, M. J. , Dinges, D. F. , & Sehgal, A. (2015). Oxalic acid and diacylglycerol 36:3 are cross‐species markers of sleep debt. Proceedings of the National Academy of Sciences of the United States of America, 112, 2569–2574. 25675494 10.1073/pnas.1417432112 PMC4345602

[jsr14438-bib-0043] Woelders, T. , Revell, V. L. , Middleton, B. , Ackermann, K. , Kayser, M. , Raynaud, F. I. , Skene, D. J. , & Hut, R. A. (2023). Machine learning estimation of human body time using metabolomic profiling. Proceedings of the National Academy of Sciences of the United States of America, 120, e2212685120. 37094145 10.1073/pnas.2212685120 PMC10161018

[jsr14438-bib-0044] Yousefi, P. D. , Suderman, M. , Langdon, R. , Whitehurst, O. , Davey Smith, G. , & Relton, C. L. (2022). DNA methylation‐based predictors of health: Applications and statistical considerations. Nature Reviews. Genetics, 23, 369–383. 10.1038/s41576-022-00465-w 35304597

[jsr14438-bib-0045] Zhang, Z. H. , Zhang, W. , Zhou, J. D. , Zhang, T. J. , Ma, J. C. , Xu, Z. J. , Lian, X. Y. , Wu, D. H. , Wen, X. M. , Deng, Z. Q. , Lin, J. , & Qian, J. (2018). Decreased SCIN expression, associated with promoter methylation, is a valuable predictor for prognosis in acute myeloid leukemia. Molecular Carcinogenesis, 57, 735–744. 29457658 10.1002/mc.22794

[jsr14438-bib-0046] Zhou, W. , Laird, P. W. , & Shen, H. (2017). Comprehensive characterization, annotation and innovative use of Infinium DNA methylation BeadChip probes. Nucleic Acids Research, 45, e22. 27924034 10.1093/nar/gkw967 PMC5389466

