# Supplementary figures and images for: Daily rhythm in DNA methylation and the effect of total sleep deprivation
Source: J Sleep Res. 2024 Dec 15;34(4):e14438. doi: 10.1111/jsr.14438 (PMC12215246; doi:10.1111/jsr.14438)

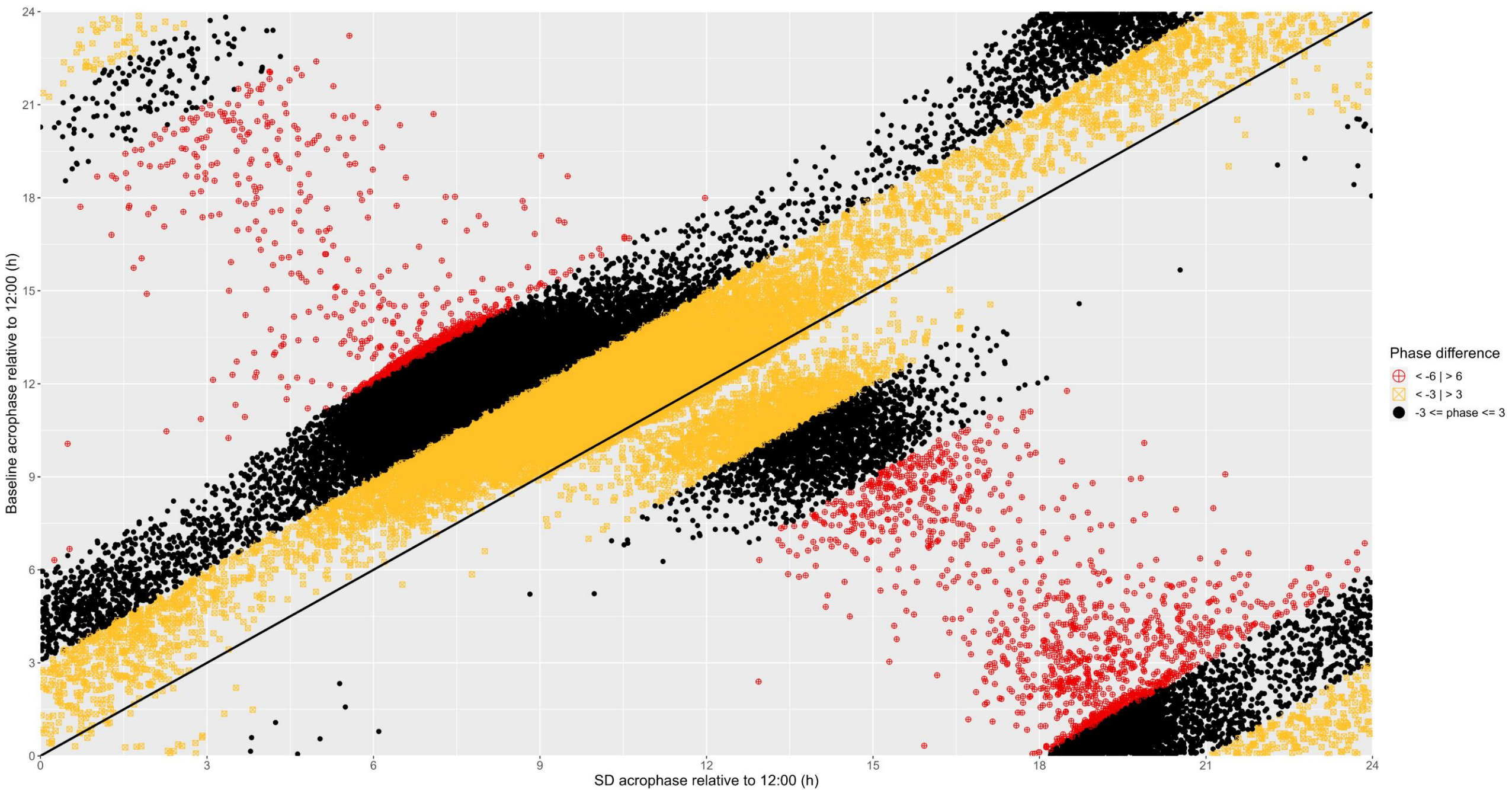

Supplement: Supplementary file 1 — FIGURE S1. A scatter plot of the Baseline acrophase versus Sleep Deprivation acrophase per rhythmic CpG site. Comparison of the acrophase (peak time) of DNAme in sleep versus sleep deprivation conditions per rhythmic CpG site. Points on the thin black line exhibit the same acrophase between conditions. [file JSR-34-e14438-s001.pdf]
