# Supplementary material for: Daily rhythm in DNA methylation and the effect of total sleep deprivation
Source: J Sleep Res. 2024 Dec 15;34(4):e14438. doi: 10.1111/jsr.14438 (PMC12215246; doi:10.1111/jsr.14438)
Supplement: Supplementary file 3 — TABLE S2. The number of CpG sites, of the 223,595 classified in the original analysis (Figure 2a), that exhibit 24‐hr rhythms post‐normalization to account for 24‐hr rhythms in white blood cell composition. [file JSR-34-e14438-s004.docx]

| Table- S2: The number of CpG sites, of the 223,595 classified in the original analysis (Figure 2A), that exhibit 24 h rhythms post normalisation to account for 24 h rhythms in white blood cell composition. | | | |
| --- | --- | --- | --- |
| Category | Rhythmic in sleep | Rhythmic in SD | % composition of WBC |
| Original non-normalised analysis (n = 15) | 100384 | 100042 | NA |
| Non-normalised analysis (n = 10) | 80230 | 79707 | NA |
| Normalised for:  total white blood cell count (n = 10) | 147245 | 143894 | 100 |
| Normalised for:  granulocytes (n = 10) | 128495 | 125576 | 54.5 |
| Normalised for:  lymphocytes (n = 10) | 153863 | 153815 | 37.3 |
| Normalised for:  Total T-cells count (n = 10) | 175911 | 175865 | 29.7 |
| Normalised for:  CD4 cells count (n = 10) | 45843 | 45429 | 19.5 |
| Normalised for:  CD4 Memory (n = 10) | 198795 | 198758 | 13.1 |
| Normalised for:  CD8 cell count (n = 10) | 189082 | 189042 | 10.2 |
| Normalised for:  monocytes (n = 10) | 73877 | 66203 | 8.8 |
| Normalised for:  CD4 naïve (n = 10) | 145463 | 145428 | 6.3 |
| Normalised for:  CD8 memory (n = 10) | 117385 | 117343 | 5.4 |
| Normalised for:  B cells count (n = 10) | 69486 | 68555 | 4.7 |
| Normalised for:  CD8 naïve (n = 10) | 153076 | 153049 | 4.7 |
| Normalised for:  natural killer/dendritic cells (n = 10) | 56423 | 53878 | 2.3 |
| Footnotes: Number of participants per dataset is displayed (n=X) for each data category. Blood cell composition data were only available for 10 participants. Percentage composition of WBC refers to the relative abundance of the named class of white blood cells averaged across the participants (n = 10) and over the time course, e.g., WBC total is the sum of all white blood cells whilst lymphocytes account for 37.3% of white blood cells in a sample. Percentage values shown are approximate and certain ‘Class’ types overlap e.g., ‘CD8 total’ comprises ‘CD8 memory’ and CD8’ naïve’. Details of flow cytometric analyses (28). | | | |
