# Supplementary material for: Daily rhythm in DNA methylation and the effect of total sleep deprivation
Source: J Sleep Res. 2024 Dec 15;34(4):e14438. doi: 10.1111/jsr.14438 (PMC12215246; doi:10.1111/jsr.14438)
Supplement: Supplementary file 4 — TABLE S3. CpG sites in the sleep deprivation condition that differed from baseline and were also classified as rhythmic Following sleep deprivation, we identified 624 sites that differed from baseline with an uncorrected p‐value of 10−4. Of these, 150 were classified as rhythmic using “compareRhythm” analysis. [file JSR-34-e14438-s002.docx]

Table S3. CpG sites in the sleep deprivation condition that differed from baseline and were also classified as rhythmic.

Following sleep deprivation, we identified 624 sites that differed from baseline with an uncorrected p-value of 10^-4. Of these, 150 were classified as rhythmic using “compareRhythm” analysis.

| Probe | category | Gene_name |
| --- | --- | --- |
| cg00299942 | change |  |
| cg00566431 | arrhy | UBE2L6 |
| cg00664093 | gain |  |
| cg00767269 | arrhy | OPA3 |
| cg00906174 | loss |  |
| cg01337197 | arrhy | A4GALT |
| cg01730181 | arrhy | NUDT5 |
| cg01885092 | arrhy | NUP62 |
| cg02058918 | arrhy | GABBR2 |
| cg02210466 | same | NDNF |
| cg02789309 | same | DUSP7 |
| cg02982753 | arrhy | MEG3 |
| cg03236534 | arrhy | ATP11A |
| cg03256465 | arrhy | JUN |
| cg03299991 | same | KCNMA1 |
| cg03306967 | arrhy |  |
| cg03339674 | arrhy | PLEKHM2 |
| cg03488275 | arrhy | DTNA |
| cg03535099 | loss | TBCD |
| cg03860530 | gain |  |
| cg04150276 | arrhy | GNPNAT1 |
| cg04251194 | same |  |
| cg04251616 | arrhy | ARHGAP15 |
| cg04468795 | arrhy | DDX50 |
| cg04499670 | arrhy | ST6GALNAC1 |
| cg04601090 | arrhy | PLEKHG5 |
| cg04659582 | same |  |
| cg04854052 | change | ARHGAP26 |
| cg04949953 | change |  |
| cg05081050 | gain | NUDT14 |
| cg05237892 | arrhy | SPATA16 |
| cg05303899 | same |  |
| cg05308867 | arrhy |  |
| cg05527782 | gain | MIR7641-2 |
| cg05599336 | same |  |
| cg05608626 | same | ZADH2 |
| cg06359253 | arrhy | DGCR2 |
| cg06787206 | arrhy |  |
| cg07055512 | arrhy | MPI |
| cg07089633 | same | DCAF4 |
| cg07138366 | loss | DGKG |
| cg07235868 | arrhy | SGSM2 |
| cg07461182 | arrhy |  |
| cg07539983 | gain | SPARC |
| cg07873926 | loss |  |
| cg07878537 | arrhy |  |
| cg07940761 | same | DEPDC5 |
| cg08199511 | arrhy | SHCBP1L |
| cg08241082 | arrhy | PTPRR |
| cg09388137 | arrhy | C3orf71 |
| cg09581065 | arrhy | RAB40B |
| cg10036732 | arrhy |  |
| cg10111146 | arrhy | ECT2 |
| cg10485101 | arrhy |  |
| cg10500218 | arrhy | IER5 |
| cg11071748 | arrhy |  |
| cg11227621 | loss | FAM36A |
| cg11260547 | arrhy | PHRF1 |
| cg11341609 | arrhy | DCTN1 |
| cg11356706 | arrhy | SCRT2 |
| cg11470399 | loss | PLK1 |
| cg11554249 | arrhy |  |
| cg11627961 | loss |  |
| cg11641222 | same | SNTB1 |
| cg11688402 | arrhy |  |
| cg11693892 | loss | ADPRHL1 |
| cg12088478 | arrhy | LINC00886 |
| cg12213135 | change |  |
| cg12587930 | arrhy | PRND |
| cg12834443 | change | TFEB |
| cg12866694 | arrhy |  |
| cg12905741 | arrhy | LINC00942 |
| cg13115957 | arrhy | C20orf202 |
| cg13529726 | loss | TOP1MT |
| cg13554213 | arrhy | C7orf20 |
| cg13776905 | loss | BPTF |
| cg13814677 | arrhy | CELSR1 |
| cg13845161 | loss | TEAD1 |
| cg13980808 | arrhy | BCAT1 |
| cg14043929 | arrhy | GPATCH2 |
| cg14146760 | same | SPX |
| cg14391730 | arrhy |  |
| cg14481619 | gain |  |
| cg14527389 | arrhy | LOC285954 |
| cg15394328 | arrhy | CLEC17A |
| cg15537902 | arrhy |  |
| cg15827532 | arrhy | LRCH4 |
| cg16575892 | arrhy | MIR378D1 |
| cg17256873 | arrhy | GPATCH2L |
| cg17316030 | same | SYNE2 |
| cg17350432 | arrhy |  |
| cg17375381 | arrhy | VGLL4 |
| cg17499345 | loss | CYR61 |
| cg17578299 | loss | NUP54 |
| cg17592292 | gain | DGKQ |
| cg17771587 | same | CSRP1 |
| cg18159478 | arrhy |  |
| cg18304601 | arrhy | CELF2 |
| cg18459342 | same | TPD52 |
| cg18706511 | gain | RCAN2 |
| cg18719863 | arrhy | C8orf37-AS1 |
| cg18801599 | change | TMEM101 |
| cg18937321 | loss | SLC6A9 |
| cg19109581 | arrhy |  |
| cg19448420 | same | SNAP23 |
| cg20068365 | loss | GMPPA |
| cg20375585 | arrhy |  |
| cg20701145 | change | DUSP16 |
| cg21161093 | arrhy | STK24 |
| cg21169115 | gain | RRH |
| cg21514086 | arrhy | SLC46A3 |
| cg21668244 | loss |  |
| cg21855109 | arrhy | TET3 |
| cg21856256 | same |  |
| cg21899743 | arrhy | MYOM2 |
| cg21975810 | arrhy | IKZF2 |
| cg22272322 | arrhy | JOSD2 |
| cg22376805 | arrhy | PROX1 |
| cg22424581 | arrhy | ARL15 |
| cg22529448 | same | ZNF559 |
| cg22542334 | loss | DDX60L |
| cg22813115 | loss | RNLS |
| cg23202291 | arrhy |  |
| cg23381598 | arrhy | RTN4R |
| cg23530463 | arrhy |  |
| cg23555187 | loss | TRIM33 |
| cg23938892 | loss | HCN3 |
| cg24005743 | same | RCN1 |
| cg24027535 | arrhy | PRIM2 |
| cg24082795 | loss | TGOLN2 |
| cg25359907 | arrhy | LOC84740 |
| cg25619607 | change | IMPACT |
| cg25772185 | gain |  |
| cg25780246 | arrhy | GIT2 |
| cg25958716 | arrhy | HIBCH |
| cg26076960 | loss | MED4 |
| cg26103205 | arrhy | ITGA2 |
| cg26360732 | same | PPP1R2 |
| cg26384678 | arrhy | TPD52 |
| cg26471781 | same | PSMD7 |
| cg26633494 | arrhy |  |
| cg27035251 | loss | JAK3 |
| cg27192986 | arrhy |  |
| cg27243623 | arrhy | COL4A3 |
| cg27276059 | change | COL12A1 |
| cg27458631 | arrhy | SF3A1 |
| cg27500961 | loss | CATSPERB |
| cg27523351 | arrhy |  |
| ch.10.176154R | loss | C10orf18 |

Footnote: Change = rhythmic and changed rhythmicity in sleep deprivation. Gain = was not rhythmic

during baseline but gained rhythmicity during sleep deprivation. Same = same rhythm in

both baseline and in sleep deprivation. Loss = rhythmic in baseline, but lost rhythmicity in

sleep deprivation. Arrhy = did not show 24 h rhythmicity in either condition.
